# Phenolic Acids in Jerusalem Artichoke (*Helianthus tuberosus* L.): Plant Organ Dependent Antioxidant Activity and Optimized Extraction from Leaves

**DOI:** 10.3390/molecules24183296

**Published:** 2019-09-10

**Authors:** Muhammad Mir Showkat, Anne Bergljot Falck-Ytter, Knut Olav Strætkvern

**Affiliations:** Department of Biotechnology, Inland Norway University of Applied Sciences (INN), Campus Hamar, Holsetgata 31, 2318 Hamar, Norway

**Keywords:** Jerusalem artichoke, chlorogenic acid, total phenolic content, ascorbic acid, antioxidant activity, yield

## Abstract

Phenolic acids including chlorogenic acids are major polyphenolic compounds found in Jerusalem artichoke (*Helianthus tuberosus* L.). The plant itself is an emerging biorefinery crop due to the inulin-rich tubers, a bioethanol feedstock, but the aerial parts represent a rich source of bioactive compounds. We have determined the level of major phenolic acids in extracts of four plant organs: tuber, leaf, flower, and stem. Employing three heating conditions (20 °C, 60 °C, and microwaving), corrected total phenolic content (TPC) was highest in the leaves (4.5–5.7 mg gallic acid equivalents g^−1^ dry substance), followed by flower (2.1–2.9), tuber (0.9–1.4), and lowest in stem extracts (0.1–0.2). A previously overlooked interference of the Folin–Ciocalteu assay, namely a signal contribution from ascorbic acid, caused overestimation of TPC in various organs ranging from 65% to 94%. Radical scavenging activity of extracts correlated significantly with TPC, both on corrected (R^2^ = 0.841) and uncorrected (R^2^ = 0.884) values. Out of the identified phenolic acids determined by quantitative HPLC-UV analysis, chlorogenic and dicaffeoylquinic acids accounted for 72–82% of corrected TPC in leaf and tuber extracts. Optimization of leaf extraction was tested in a 2^3^-factorial Central Composite Face (CCF) design. Temperature was the most important model term, and a solvent strength of less than 50% ethanol promoted the highest TPC yields. Further developments in extraction processing of crop residues may open avenues for improving the utilization of Jerusalem artichoke in valuable products.

## 1. Introduction

Among biologically protective polyphenols, phenolic acids are widely distributed in plants, and have antioxidant capacity by neutralizing radicals by donating or accepting electrons. Major classes of phenolic acids comprise carbon chains of C6+C1 (hydroxybenzoic acids, HBA) and of C6+C3 (hydroxycinnamic acids, HCA), where C6 is the phenyl ring and C3 the propanoid moiety of various *trans*-cinnamic acids (caffeic, *p*-coumaric, and ferulic acid). Jerusalem artichoke (*Helianthus tuberosus* L.) is a perennial tuberous plant for which the content of phenolic substances has been described by several authors. Tchone and colleagues [1] identified 22 phenolic compounds in methanolic extracts of tubers; salicylic acid ranked highest of the HBAs, and highest of the HCAs was chlorogenic acid (CQA), an ester of caffeic acid and quinic acid. Within the family of caffeoyl quinic acids, the hydroxyl groups of quinic acid can be esterified with caffeic acid in multiple positions (1, 3, 4, 5) to produce mono-, di-, and even tricaffeoyl isomers. The leaves, in particular, contain a broad specter of mono- and dicaffeoyl esters of CQA; more than 30 variants were identified by LC-MS [2]. Out of these, the 3-O-caffeoylquinic acid (3-CQA) isomer and two dicaffeoyl esters of quinic acid (1,5- and 3,5-diCQA) were dominating. Additionally, Chen and colleagues [3] also observed high levels of the 4,5-diCQA isomer in leaves. In the tubers of Jerusalem artichoke, three isomers of CQA and four isomers of dicaffeoylquinic acids have been identified [4]. 

Due to the diversity of the CQA isomers, their abundance in solvent extracts and corresponding antioxidant activity depend on the strength and polarity of the solvent system used. Exploratory extractions of polyphenols can be lengthy, stretching from several hours up to overnight, followed by classical sequential extractions and separations into more non-polar solvents. The most abundant phenolic acid obtained from leaves extracted by ethyl acetate was 1,5-diCQA (104 mg/g dry substance (DS), or 39%), while 3-CQA (74 mg/g DS or 55%) dominated in the *n*-butanol fraction extracts [5]. Ethyl acetate extracts also showed the highest radical scavenging activity (SC_50_). Polar solvent systems, e.g., ethanol [3,5,6,7,8] or methanol [2,4,9,10], are frequently used for the initial extraction of polyphenols from leaf and tuber material. Moreover, to improve yields, extractions are sometimes intensified with ultrasound [2,7], heat [3,9], or pressure [4]. For example, Petrova and colleagues [7] made extracts of Jerusalem artichoke flowers in 95%, 80%, and 70% ethanol applying an ultrasound bath, which produced the highest total phenolic content (TPC; 17.4 mg gallic acid equivalents (GAE) per gram fresh weight) in 70% ethanol. In a high-yielding preparative method, the 3-CQA, an isomer of considerable commercial value, was purified from leaves. The protocol contained repeated extractions in methanol (60% at 60 °C and 3 × 30 min), followed by column adsorption on a polar resin eluted with sequential flushes of 60–90% ethanol, resulting in a more than 5-fold enrichment and 89% yield of 3-CQA [9].

The potential commercial value of polyphenolic substances in Jerusalem artichoke, including the CQAs, is connected to the many reported beneficial bioactivities, besides antioxidants, found in tuber and aerial organs (leaf, flower, and stems), reviewed by Lim [11] and Yang and colleagues [12]. The phenolic content of ethanol extracts of tubers and leaves elicited the effects observed in anti-inflammatory and cytotoxic [13] as well as antifungal [6] bioassays. Although tubers of Jerusalem artichoke are considered both a valuable food and energy (inulin) crop [14], its cultivation in northern countries remains limited [15,16]. From a bio-economy perspective, finding better uses also for the top residues or aerial parts other than as animal feed may increase the overall value of the crop. Considering the usefulness and biological potential of phenolic acids, the objectives of the study were to assess the distribution and antioxidant activity of the major phenolic acids throughout the whole plant (tuber, stem, leaf, and flower). We also wanted to chart the boundaries of critical variables (holding time, solvent strength, and temperature) for ethanol extraction of phenolic acids from leaf material, starting out investigating whether thermal intensification could improve yields.

## 2. Results

### 2.1. Phenolic Acids Extracted under Different Conditions

**Figure 1:**
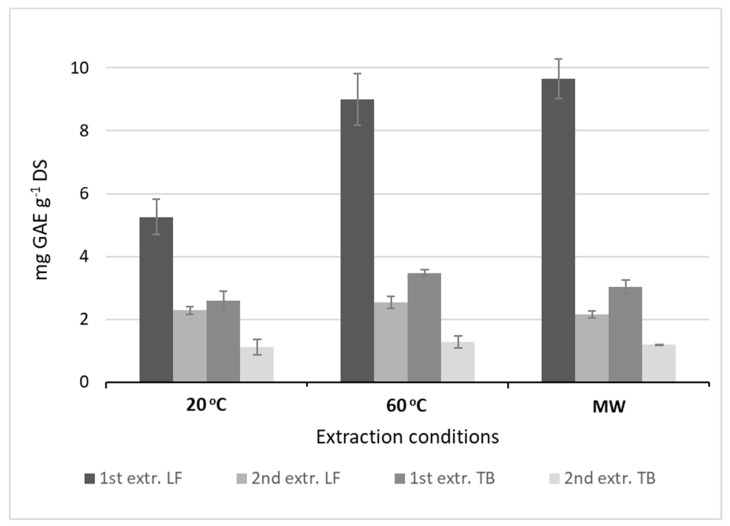
Effect of two-step extraction (1st, 2nd) and different extraction conditions on the yield of total phenolic content (TPC; mg gallic acid equivalents per g dry substance) from Jerusalem artichoke leaf (LF) and tuber (TB). Bars represent the mean (±SD) of replicate extractions (*n* = 2). Significant differences were observed between the extraction steps, and the extraction methods 20 °C, 60 °C, and microwaving (MW) (F_1, 2_ = 18.51, *p* < 0.05).

For initial screening of conditions, a two-step extraction from leaves and tubers were compared for the overall yield of total phenolics. In the second step, the residue from the first step was re-extracted with a new volume of fresh solvent. Leaf extracts contained double to threefold more phenolics than the tuber extracts (Figure 1). For the same extractions, the effect of incubation temperature (20 °C and 60 °C) and microwaving (MW) was also tested. Temperature had a significant effect on extraction from room temperature up to 60 °C; only a marginal effect was obtained with microwave-assisted extraction. First-step extraction from leaves gave 70% of the combined yield at room temperature, and increased further to 78% at 60 °C and 82% at microwave conditions. In the continued study, all three temperature conditions were used, but applying only single-step extraction due to the significantly higher yield obtained.

### 2.2. Distribution of Total Phenolic Content in the Whole Plant

**Figure 2:**
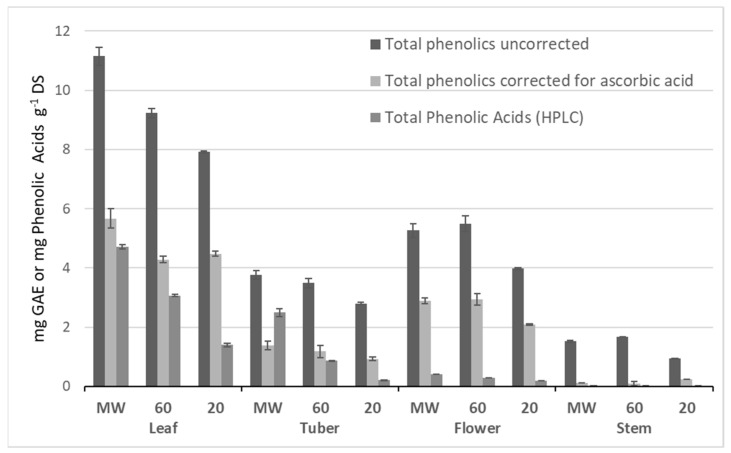
Distribution of total phenolic content (TPC) in Jerusalem artichoke plant organs measured as mg gallic acid equivalents (GAE) per g dry substance (DS) in ethanol extracts. Plant organ extracts prepared at three heating levels (MW, microwaving; 60, 60 °C; and 20, 20 °C). TPC is shown both uncorrected and corrected for ascorbic acid contribution. Also shown is the sum of major phenolic acids (mg g^−1^ DS) determined by HPLC-UV. Bars represent the mean (±SD) of replicate extractions (*n* = 2).

Figure 2 shows the distribution of total extracted phenolics (mg GAE g^−1^ DS) in various organs of the plant; leaf (7.9–11.1), flower (4.0–5.3), tuber (2.8–3.8), and stem (0.9–1.7). It should be noted that the ascorbic acid content of vegetables [17], including that of Jerusalem artichoke [11], also contributes to the response in the total phenolics assay by reducing the Folin–Ciocalteu reagent. The basic Folin–Ciocalteu (F–C) assay was modified accordingly [17,18] and corrected TPC values displayed (Figure 2). Still, the corrected TPC values showed that ethanol extract of leaves (4.5–5.7) contain higher phenolic acid content than flower (2.1–2.9), tuber (0.9–1.4), and stem (0.1–0.2) extracts. Stem extracts showed very low phenolic acid content compared to the other plant organs. Thus, ascorbic acid appeared to be a major contributor to uncorrected TPC depending on the various extraction conditions, up to 65% in flowers, 68% in leaves, 75% in tubers, and 94% in stems.

### 2.3. Antioxidant Activity 

**Figure 3:**
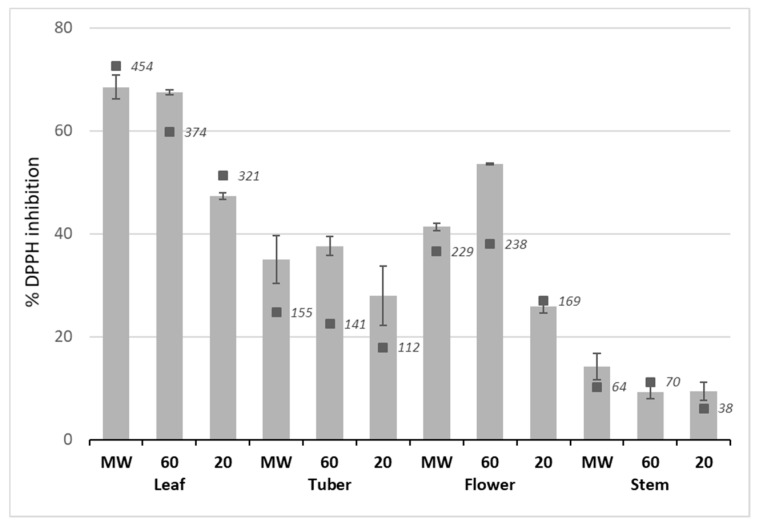
Antioxidant capacity in ethanol extracts of Jerusalem artichoke as percent inhibition of DPPH. Plant organ extracts prepared at three heating levels (MW, microwaving; 60, 60 °C; and 20, 20 °C). Bars (grey) represent the mean (±SD) of replicate of extractions (*n* = 2). Black squares and italicized numbers represent the extract concentration of total phenolics (µg mL^−1^) uncorrected.

The experiment was carried out to compare the radical scavenging activity of the phenolic acids. The calculated percent inhibition is presented in Figure 3. Ethanol extract of leaves from microwave heating and from 60 °C showed the strongest radical scavenging activity, followed by flower and tuber, whereas stem extracts demonstrated the lowest inhibition capacity. 

**Figure 4:**
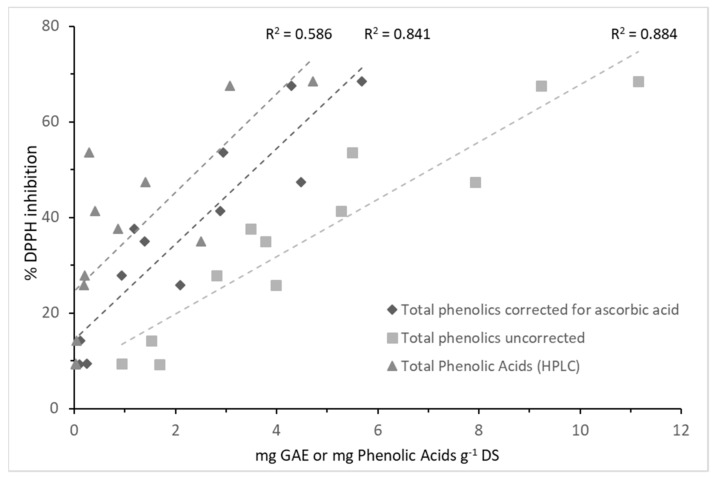
Scatter plots of the radical scavenging activity (% inhibition of DPPH) in ethanol extracts of Jerusalem artichoke plant organs, versus uncorrected and corrected total phenolic content, and the total phenolic acids analyzed by HPLC-UV. Coefficient of determination (R^2^) for the three plots are 0.884, 0.841, and 0.586, respectively.

Given the parallel responses, a correlation plot of TPC values and radical scavenging activity was analyzed (Figure 4). Significant positive linear correlations were evident for percentage of inhibition, and for uncorrected (R^2^ = 0.884) and corrected total phenolics (R^2^ = 0.841). Thus, applying the correction for ascorbic acid caused the steeper slope of the correlation. 

### 2.4. Identification and Quantitation of Major Phenolic Acids 

**Figure 5:**
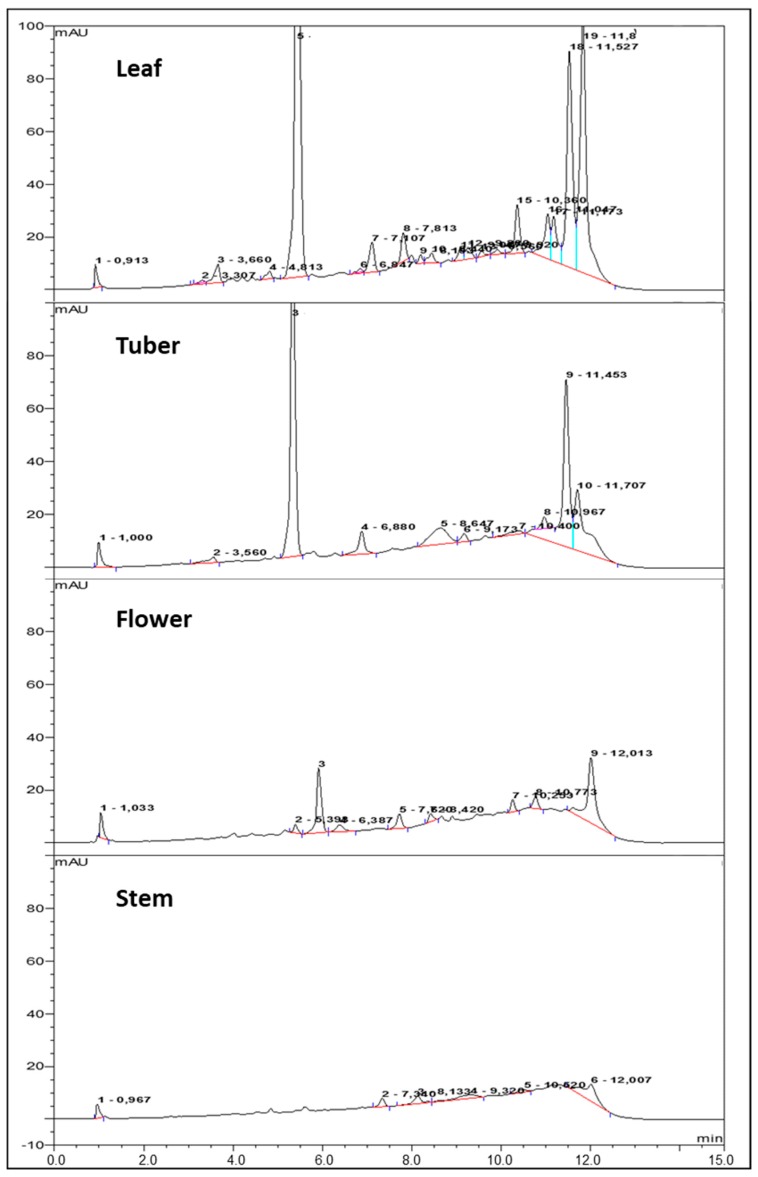
Response vs. acquisition time profiles from HPLC-UV analysis of microwave (MW) ethanol extracts of Jerusalem artichoke leaf, tuber, flower, and stem. The column used was a KINETEX 5 μm C18 100 mm × 4.6 mm (25 °C) and absorbance measured at 330 nm. The mobile phase (1.5 mL min^−1^) was composed of water with 0.1% TFA (A) and Acetonitrile (B). The initial A/B ratio was 95/5, increasing to 80/20 over 10 min and held for 1 min during the analysis, before A/B again was returned to 95/5.

By use of HPLC-UV, the gross composition of phenolic acids in extracts was determined by comparison to retention time (RT) of reference standards. Based on the detailed study by Jaiswal and colleagues [2], these references included CQA, CA, PQA, FA, 1,5-diCQA, and 3,5-diCQA. Ethanol extract of leaves, flowers, and tubers resolved multiple compound peaks of varied height between 3 and 12 min (Figure 5), while in extracts of stems only minor peaks were detected. The expected RT after sample spiking (*n* = 3) were 5.6 min (±0.4) for CQA, 6 min (±0.3) for CA, 8.5 min (±0.6) for PQA, 11.5 min (±0.6) for 1,5-diCQA, and 11.6 min (±0.3) for 3,5-diCQA. Identification of FA (RT = 8 min) was attempted but did not provide any detectable signal, and was therefore excluded for further quantification. Peaks corresponding to CQA, 1,5-DiCQA, and 3,5-DiCQA were the most pronounced in chromatograms of leaves, tubers, and flowers. Twin peaks at ca. 11 min corresponding to 3,5 and 1,5-diCQA [2] were highly typical of the leaf samples. 

Table 1 provides the composition of the plant organs at various extraction conditions. The highest CQA content was in leaves; 2.5 (60 °C) and 2.2 mg g^−1^ DS (microwave), followed by 1.48 mg g^−1^ DS (microwave) in tubers. CA and PQA were detected in leaves, tubers and flowers, but at negligible levels. As also seen in the chromatograms, the peaks corresponding to dicaffeoyl isomers 1,5 and 3,5-diCQA were highest in leaf microwave extracts (1.07 and 1.39 mg g^−1^ DS, respectively), whereas in tubers and flowers only found at low levels.

The sum of quantified peaks in each extract was compared to the extract TPC corrected for ascorbic acid (Table 1 and Figure 4); in leaf extracts at microwave and 60 °C, 82.7% and 71.5% of the TPC were accounted for by HPLC-UV analysis, respectively. In tuber extracts also, the peak estimation corresponded to the TPC levels fairly well. The sums of quantified peaks within each extract of organ and heat treatment were correlated to the corresponding DPPH inhibition activity (Figure 4). However, this correlation resulted in a lower coefficient of determination (R^2^ = 0.59); less than 60% of the HPLC-UV data are explained. This may indicate that non-identified phenolic compounds contributed to the DPPH response. 

### 2.5. Factorial Design of Extraction Conditions

The screening of critical variables in extracting total phenolics from leaves was set up with a full factorial design employing a Central Composite Face (CCF) model. The coded variables and the corresponding responses are given in Table 6. The model coefficients (β) were calculated and used to quantify the effect of each variable on the extraction response (Table 2). The significance of each term in the model was determined by a variance test (ANOVA), where a *p*-value lower than 0.05 indicates that the variable contributes significantly to the response. The largest contribution came from temperature (X_2_). The order of significance of the orthogonal variables on extraction yield was, according to the *p*-values, X_2_ > X_1_ > X_3_. No interaction terms (X_i_X_j_) were determined. To improve a linear model into a prediction tool additional curvature terms can be added. Thus, the quality of the square terms (X_i_^2^) were analyzed. According to the *p*-values, only squared temperature provided significant curvature, while square terms of ethanol concentration and time were insignificant (Table 2). The model was not refined further. 

Analysis of variance showed that the regression is statistically significant (*p* < 0.05) for the observed response (Table 3). The fact that the model F-value (18.29) exceeds the critical value (F_6,25_ = 2.49) for the 5% significance level indicates that treatment differences are highly significant. Thus, the established regression adequately represents the observed data. The coefficients of determination (R^2^ and R^2^ adjusted) states the validity of the model. The values, 0.814 and 0.770, respectively, are of a magnitude that confirms a good correlation between model and experimental data, explaining about 80% of the total variance in the data. A further underpinning that the model is compatible with the data comes from variance analysis of “lack of fit” relative to “pure error”. The corresponding F-value (0.397) is well below the critical value (F_6,19_ = 2.63) and the variance is therefore not significant (*p* > 0.05).

Supported by the statistical analysis, an empirical model was set up (Equation (1)) to describe the change in total phenolic acid yield (Y_TP_, mg GAE g^−1^ DS) as a function of ethanol strength (E), temperature (T), and holding time (t):

Y_TP_ = −6.80 − 0.59E + 0.94T − 0.57t − 0.73T^2^. (1)

**Figure 6:**
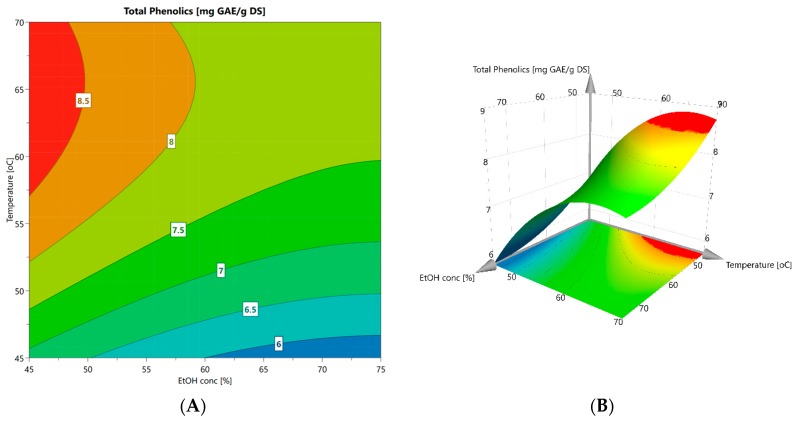
Contour (**A**) and response surface (**B**) plots at t = 60 min for extraction of phenolic acids from leaf material.

Figure 6 shows a contour plot and a response surface plot at t = 60 min that reflects the variation in the most significant variables, temperature and ethanol concentration. The plots highlight a curved area (orange-red) along the temperature axis where conditions predict a yield of total phenolic equal to or higher than 8.0 mg GAE g^−1^ DS, with a likelihood of 95%. Within this window of operation, the following experimental conditions were chosen for evaluating the model’s predictability: An ethanol concentration of 48%, temperature of 57 °C, and extraction time of 60 min. Since time (t) is a negative term in the empirical model, the low-end range of holding time was selected. The outcome of the model-set extractions was compared to the conditions (60%, 60 °C, and 60 min) used as standard for extraction from the various plant organs, including leaves. Table 4 shows a comparison of the predicted and experimental extraction yields. Interestingly, the experimental conditions resulted in yields that were about 14% higher than predicted, including the model error. Moreover, no significant difference in the yield between the model-set and standard conditions indicates that there probably is a wider and more robust window of operation than predicted by the model. This assumption is supported by the total phenolic yields from leaf already reported in Figure 1 and Figure 2 (about 9 mg GAE g^−1^ DS).

## 3. Discussion

The Jerusalem artichoke has been proposed as a biorefinery crop due to the multipurpose uses of the plant [12,19,20]. The inulin-rich tubers are the main crop, mainly for food use and for bioethanol production. Well documented in the literature, the aerial parts represent a rich source of bioactive constituents including phenolic acids, and therefore logical targets for such value-added utilization. However, as indicated by Johansson and colleagues [14], suitable extraction procedures must be developed for utilization of the whole plant to be economically viable, not only as an energy crop. To work out an approach for biomass utilization, we undertook an assessment of total phenolic content (TPC) in various plant organs. The genotype “Dagnøytral” is an early variety of Jerusalem artichoke, developed for growth in the Nordic climate [15]. When fully grown in October, it reaches a height of about 2.5 m. Mid-season, the leaves can make up about 40% of the plant dry mass, while the stalk grows thicker and at the time of harvest in November dominates the dry mass of aerial parts [15,19]. 

Using a polar solvent extraction from dried material, both leaves and flowers measured high in TPC, while tuber and stem extracts were low or almost absent. This distribution of protective constituents may reflect that plant surfaces most readily exposed to sunlight are prone to accumulating antioxidant compounds. However, various environmental factors and stage of maturity can affect the accumulation of phenolics in plants [3,21]. 

Similar to numerous studies on phenolic content in plant material, the Folin–Ciocalteu (F–C) assay was used for TPC analysis. But in this study, we employed the modification suggested by Isabelle and colleagues [17] on correcting for the likely contribution from ascorbic acid (e.g., vitamin C). Ascorbic acid reacts more rapidly with the F–C reagent than the slow-reacting phenolic acids, and thus the reducing reaction of the latter can be distinguished and quantitated to obtain a more accurate total phenolic content [18]. This assay modification resulted in significantly lower TPC in all extract types—in some cases more than 50%. Without correcting for the ascorbic acid contribution, the total phenolic acid content will unintentionally be overestimated unless quantitated by hyphenated high-resolution methods. Despite overestimation, the correlation between the radical scavenging activity (% DPPH inhibition) and the TPC values, both raw and corrected, from all plant organs and extract types, were linear and highly significant. A similar correlation between antioxidant activity assays (DDPH and FRAP) and TPC (uncorrected) was observed in extracts of five edible flowers including those of Jerusalem artichoke. On the other hand, the flavonoid content in flowers did not correlate to the same assays [7]. Another antioxidant assay, the oxygen radical absorbance capacity (ORAC) of acetone extracts made from 66 vegetables correlated with the same good linearity (R^2^ = 0.81) to corrected TPC values [17]. Thus, given the substantial positive correlation it is likely that the phenolic acids in our extracts are responsible for the observed antioxidant activity.

Studies reporting on the detailed composition of phenolic acids in Jerusalem artichoke leaves [2,3,5], the plant organ of highest abundance, and in tubers [4], all agree on the prevalence of 3-CQA and the various dicaffeoyl isomers (i.e., 1,5, 3,5, and 4,5). Our conditional data seem to agree with this picture. In our assessment, the reference standards for 1,5 and 3,5-dicaffeoylquinic acid corresponded well with the major twin peak (RT = 11.5–11.6 min), however the presence of additional isomers cannot be excluded. Even with minor unidentified peaks, the quantitative HPLC-UV analysis of leaf and tuber extracts accounted for the more than seventy percent of the corrected TPC. The actual amount of phenolic acids in the leaf, though, appears to vary ten-fold (24 [3]–267 [5] mg g^−1^ DS), depending on the solvent system and extraction conditions. Growth conditions, harvest time, and plant variety may also affect the yield. With the “Dagnøytral” variety used in the current study, leaf yields (4–6 mg g^−1^ DS) are modest in comparison, but re-extraction or prolonged holding time may increase yields about 20%. The overall yield of TPC increased significantly from a standard room temperature extraction under stirring via heated stirring at 60 °C, to intensified short-term microwave extraction. Solvent polarity differentiates on the extractability [5] but phenolic acids in general dissolves well into polar solutions like aqueous alcohols (MetOH, EtOH). Taking into account the use of rational, high-yielding, and environmentally friendly extraction procedures, ethanol is a preferred solvent [22]. This was also the conclusion of a study comparing extraction of antioxidants from herbs and spices into acetone (80%), ethanol (80%), methanol (80%), and distilled water [23]. Water only, however, contrasts the apparent positive effects of elevated temperature and polar solvent, when used as solvent in a low-temperature high-pressure (LTHP) extraction with leaves. Compared to stirring, reflux, and autoclave extractions in water, LTHP resulted in substantial polyphenol yield (56.7 mg GAE g^−1^ DS) and antioxidant activity [24]. 

In our pursuit of simple and efficient means to obtain bioactive phenolic acids from leaf material, an optimization of extraction in aqueous EtOH and under heated stirring, appeared as the most rational approach. Thus, temperature, EtOH strength, and holding time were significant variables to test, and a CCF model allowed for curvature to the prediction surface. The outcome of the factorial design resulted in a model with a high degree of significance from which reliable predictions were made. When comparing the model set conditions (48%, 57 °C, and 60 min) to the reference conditions (60%, 60 °C, and 60 min), the corrected TPC values were undistinguishable, indicating the window of operation is quite wide and robust. Interestingly, different from several previous studies employing 70–90% aqueous EtOH, a solvent strength of 50% and below promoted the highest total phenolic yield from leaves. The high relative polarity of this solvent makes extraction of polar substances more likely, i.e., phenolic acids, leaving the less polar compounds in the leaf behind. These substances constitute bioactivities of considerable interest [6,10,25]. The simple heated-stirring method has potential for scaling-up to obtain crude extracts of phenolics from bulk biomass. Further refinements of the recovered phenolic acids are possible, e.g., highly purified chlorogenic acid (3-CQA) from *H. tuberosus* was successfully demonstrated by column adsorption to a macroporous polar resin, suitable for large-scale manufacturing [9]. 

## 4. Materials and Methods 

### 4.1. Chemicals 

The chemicals including Gallic acid (G7384), ascorbic acid (20150.184), Folin–Ciocalteu phenol reagent 2N (F9252), α,α-diphenyl-β-picrylhydrazyl (DPPH) (D9132), chlorogenic acid (C3878), *p*-coumaric acid (C9008), caffeic acid (C0625), ferulic acid (1270311), 1,5-dicaffeoylquinic acid (16917), and 3,5-dicaffeoylquinic acid (SMB00131) were purchased from Sigma Aldrich (St. Louis, MO, USA).

All other lab chemicals not listed here were of analytical grade and obtained from general lab suppliers (i.e., VWR Norge). Absolute ethanol (96%) for assay purposes was purchased from Kemetyl Norge AS (Trollåsen, Norway). For extraction use, a technical grade, rectified fraction of ethanol (80% *w*/*v*) was obtained from a local distillery process (HOFF SA, Gjøvik).

### 4.2. Plant Material

Fresh tubers, stems, leaves, and flowers of Jerusalem artichoke were collected in late September from a 20 ha growth field at Fredheim farm (60.715° N, 11.127° E), Stange, Norway. The plant material was obtained from three individual full-grown plants (>2 m) spaced at least 5 m apart. The plants were of the Norwegian variety “Dagnøytral” and in their second growth season. Plant organs were sorted on site, then sealed and stored in the dark at 4 °C until treatment.

### 4.3. Pre-Treatment and Extraction 

Plant organs were cut into pieces (<1 cm × 1 cm), or sliced (tubers, 2 mm), before freezing on plastic trays and subsequently freeze-dried (CHRIST ALPHA 1-4 freeze dryer; coil temperature −50 °C, vacuum 0.30 mbar). After freeze-drying, the material was crushed and the powder sifted manually through sieves of mesh sizes 1 and 0.5 mm. Extraction in ethanol was used for all samples and three temperature conditions were applied. A standard extraction volume was 30 mL of solvent per one gram dry solids (DS) sample.

#### 4.3.1. Microwave-Assisted Extraction 

A Teflon digestion vessel (BOLA A240-06, Bohlender GmbH, Grünsfeld, Germany) recommended for temperatures up to 150 °C was used. Sample and solvent (30 mL) were gently mixed into an inner lining before the vessel was sealed and irradiated for 2 min at 120 W in a kitchen MW unit (Samsung-ME82V). The vessel was cooled before the crude extract was recovered. The inner lining was carefully cleaned between each sample extraction. 

#### 4.3.2. Heated Stirring Extraction 

Samples with solvent (30 mL) were mixed in sealed Erlenmeyer flasks (100 mL), and placed on an orbital shaker (ca. 80–100 rpm) inside a laboratory incubator set at 60 °C. Retrieved after the designated time (normally 60 min), flasks were cooled before the crude extract was recovered.

#### 4.3.3. Room Temperature Stirring Extraction 

Samples with solvent (30 mL) were mixed in sealed Erlenmeyer flasks (100 mL), placed on an orbital shaker (ca. 80–100 rpm), and left at room temperature (approximate 20 °C). After the designated holding time (normally 60 min), the crude extract was recovered. 

#### 4.3.4. Post-Extraction Treatments

The mixture of sample material and solvent was centrifuged at 5000 *g* for 15 min and 4 °C (Beckman Coulter Allegra TM 25R). The supernatant was carefully decanted and filtrated free of any remaining particles through a Whatman No.1 glass microfiber filter (4.7 cm) on a Büchner funnel using vacuum suction. Clarified extracts were stored cold in sealed 50 mL Falcon tubes until analysis.

### 4.4. Corrected Total Phenolic Content 

Total phenolic content (TPC) of extracts was analyzed using the Folin–Ciocalteu (F–C) assay applying the correction for ascorbic acid (AA) as introduced by Isabelle and colleagues [17], and Sánchez-Rangel and colleagues [18]. Na_2_CO_3_ solution was prepared by dissolving 20 g of anhydrous Na_2_CO_3_ in 80 mL of water and bringing the mixture to a boil. After cooling, a few crystals of Na_2_CO_3_ were added. After 24 h, the solution was filled up to 100 mL and clarified through Whatman No.1 filter paper. For the reaction, 1.58 mL of water was added to 20 μL of the standard or sample solution, followed by addition of 100 μL of the F–C reagent. After 3 min of incubation at room temperature, the solution absorbance was read at 765 nm (UV-1601 spectrophotometer, Shimadzu Europa GmbH, Duisburg, Germany) to determine the reagent reaction with ascorbic acid. A standard curve (R^2^ = 0.999) of ascorbic acid (AA) was prepared by using solutions of 0, 0.25, 0.5, 1, and 2 g mL^−1^. Next, 300 μL of the Na_2_CO_3_ solution was added to the reaction mixture and incubated further for 2 h at room temperature. The total absorbance of the reaction including that of the phenolic content was determined at 765 nm. A standard curve (R^2^ = 0.999) was prepared by using gallic acid solutions of 0, 50, 100, 150, 250, and 500 mg L^−1^.

A corrected TPC value was obtained by first estimating the AA content (mg mL^−1^) from the standard curve. Hence, it was established that 1 mg of AA had a reducing activity equivalent to 0.571 mg of GA, which in turn is used as a conversion factor expressing the AA reducing activity in the extract. Accordingly, the total AA content in the plant material extract was multiplied by 0.571, and the value subtracted from the TPC obtained after 2 h with the gallic acid standard curve, i.e.,:

Corrected TPC (mg GA/mL) = TPC − AA reducing activity. (2)

### 4.5. Antioxidant Activity 

The radical scavenging activity of phenolic acids was determined by the method of Sharma and Bath [26] measuring the samples’ quenching capacity of radicals generated by the DPPH reagent. The assay was carried out in a microplate format using a FLUOstar OPTIMA microplate reader (BMG LABTECH). Standard solutions of chlorogenic acid and ascorbic acid were used at 0.1 mg mL^−1^. Each microwell comprised of 100 μL of 60% EtOH, 2 μL of the standard phenolic acid solution, or an unknown sample and 100 μL of DPPH solution (50 μM). The negative control was 100 μL of DPPH solution and 100 μL of 60% EtOH. All samples were assayed in triplicate. The blank was of 200 μL 60% EtOH. Absorbance (Abs.) was measured automatically at 520 nm every minute for 30 min. The change in assay absorbance at the endpoint relative to the negative control was used to calculate the percentage of inhibition or quenching, by the sample according to the formula

% DPPH inhibition = [(Abs. of control − Abs. of sample)/(Abs. of control)] × 100.(3)

### 4.6. Identification and Quantification of Phenolic Acids

The composition of extracts was determined by HPLC-UV comparing retention times of chlorogenic acid (CQA), caffeic acid (CA), *p*-coumaric acid (PQA), ferulic acid (FA), 1,5-dicaffeoylquinic acid (1.5 diCQA), and 3,5-dicaffeoylquinic acid (3,5 diCQA). The UltiMate3000 LC-UV system (Thermo Scientific) was used, fitted with a KINETEX 5µm C18 column (100 mm × 4.6 mm, Phenomenex) held at 25 °C. Peak data acquisition was made at 330 nm and 1.5 mL min^−1^. The mobile phase was composed of 95% trifluoroacetic acid (TFA, 0.1%) and 5% Acetonitrile (ACN). Standard curves of reference compounds were prepared by analyzing solutions at 0.01, 0.05, and 0.1 mgL^−1^.

### 4.7. Statistical Analysis

Analysis of variation (ANOVA) was performed on analytical values to test whether there was a significant difference between treatments and samples at a level of significance *p* < 0.05. ANOVA was applied to TPC analysis and antioxidant activity analysis. 

### 4.8. Experimental Factorial Design in Extraction Conditions

To investigate the conditions giving the highest extraction yield of total phenolics from leaves, three experimental variables (ethanol concentration, temperature, and holding time) were screened in a 2^3^-structured factorial approach using the quadratic Central Composite Face (CCF) model. The CCF design consisted of eight corner conditions (run 1–8), six star points on the cube faces (run 9–14), and a center point in triplicate (run 15–17), representing 15 unique conditions (Table 6). All extractions were carried out in technical replicates, resulting in 34 observations from the whole experiment. The MODDE 12.1 Pro (MKS Umetrics AB, Umeå, Sweden) software was used to generate the matrix, calculate regression coefficients, evaluate significance of variables, statistical analysis, and create charts for interpretation. In the statistical calculation, the real variable values were coded as dimensionless values (X_i_). Center points allowed for calculation of experimental error and reproducibility. A response surface was determined by multiple linear regression (MLR) from coded table and matrix values (Table 5 and Table 6). Results from the MLR treatment were obtained with 95% confidence interval (*p* < 0.05). A non-linear polynomial regression model was assumed according to the equation

y = β_0_ + β_1_x_1_+ β_2_x_2_ + β_3_x_3_ + β_11_x^2^_1_ + β_22_x^2^_2_ + β_33_x^2^_3_... + ε, (4)

where β_0_ is the constant (intercept) term, β_i_ are the linear regression coefficients (main effects) of each variable x_i_, β_ii_ are the squared coefficients, and ε is the residual term. The research hypotheses were β_i_ and β_ii_ ≠ 0. The suitability of the model was tested by analysis of variance at the 5% level using a Fischer F-test. Coefficients of determination (R^2^) were evaluated for the linear and squared models. 

## 5. Conclusions

The current study has made an overall assessment of total phenolic content in the Jerusalem artichoke plant, taking into careful consideration that TPC values are easily overestimated. Correcting for ascorbic acid contribution, TPC was highest in leaves and flowers, while tubers in the studied variety are low in TPC. The main phenolic acids are chlorogenic (3-CQA) and isomers of dicaffeoylquinic acid, tentatively identified as the 1,5 and 3,5-isomers. Radical scavenging activity correlated positively with the TPC extracted in various plant organs, confirming the antioxidant bioactivity of phenolic acids. A simplified extraction method for leaves into aqueous EtOH demonstrated that predictable yields of TPC are attainable. Evaluated as a biorefinery crop, leaves of Jerusalem artichoke should be considered a valuable side crop at the time of tuber harvest. Further research into processing of the biomass will be helpful for exploiting the commercially interesting phenolic acids; as natural antioxidants in foods, in pharmaceutical and cosmetology applications, and as fungicide [6]. From the same biomass, added value can arise by developing differential solvent extraction procedures that could also fractionate the cytotoxic bioactivities [10,25]. 

## Figures and Tables

**Table 1:** Plant organ concentrations (mg g^−1^ DS) of main phenolic acids in Jerusalem artichoke, assessed by HPLC-UV analysis of chlorogenic acid (CQA), *p*-coumaric acid (PQA), caffeic acid (CA), and the dicaffeoyl isomers 1,5-diCQA and 3,5-diCQA. Extracts are grouped according to organ and heating conditions (MW or °C).

Sample	Condition	CQA	PQA	CA	1,5 diCQA	3,5 diCQA	Sum	% of TPC corr.
Leaves	MW 60 20	2.23 2.52 0.91	0.02 0.02 0.10	0.00 0.00 0.00	1.07 0.19 0.38	1.39 0.34 0.01	4.71 3.07 1.40	82.7 71.5 31.2
Tuber	MW 60 20	1.48 0.65 0.13	0.00 0.00 0.00	0.07 0.08 0.08	0.13 0.14 0.00	0.82 0.00 0.00	2.50 0.87 0.21	178.6 72.3 23.1
Flower	MW 60 20	0.14 0.20 0.04	0.03 0.03 0.03	0.06 0.06 0.07	0.17 0.00 0.01	0.01 0.00 0.04	0.41 0.29 0.19	14.1 10.0 8.9
Stem	MW 60 20	0.01 0.01 0.00	0.00 0.01 0.02	0.00 0.00 0.00	0.01 0.00 0.00	0.02 0.00 0.00	0.04 0.02 0.02	36.1 19.0 10.5

**Table 2:** Calculated regression coefficients for terms in the multiple linear regression model (Equation (4)).

Model Term	Coefficient (β)	*p*-Value
Intercept (β_o_)	6.798	<0.001
Ethanol conc. (X_1_)	−0.589	<0.001
Temperature (X_2_)	0.937	<0.001
Holding time (X_3_)	−0.567	0.001
X_1_X_1_	0.281	0.250
X_2_X_2_	−0.730	0.004
X_3_X_3_	0.301	0.219

**Table 3:** ANOVA for the regression model on extraction of phenolic acids from leaf material.

Source of Variation	Sum Quadratic	DF	Mean Quadratic (Variance)	F-Value	*p*	SD
Total corrected	43.21	31	1.39			1.18
Regression	35.20	6	5.87	18.29	<0.001	2.42
Residual	8.02	25	0.321			0.566
Lack of fit (model error)	0.894	6	0.149	0.397	0.872	0.386
Pure error (replicate error)	7.12	19	0.375			0.612
R^2^	0.814					
R^2^ adjusted	0.770					

**Table 4:** Outcome of extraction yields (mg GAE g^−1^ DS) of total phenolics from leaf material compared with the empirical model. Corrected TPC values were also assayed for the experiments.

Response	Predicted from Model (Equation (1))	Model-set Conditions (*n* = 3)	Standard Conditions (*n* = 2)
TPC uncorr. (±SD)	8.28 ± 0.6	9.42 ± 0.06	9.36 ± 0.06
TPC corrected (±SD)	n.d.	6.08 ± 0.06	6.00 ± 0.04

**Table 5:** Coding and levels of variables considered in optimizing extraction yield of phenolic acids.

Variable	Factor	Levels		
		−1	0	1
Ethanol strength (%)	X_1_	45	60	75
Temperature (°C)	X_2_	45	58	70 *
Holding time (min)	X_3_	60	120	180

* High-end temperature was set below the boiling point of ethanol to avoid unintended evaporation.

**Table 6:** Full factorial design for the Central Composite Face (CCF) model with experimental responses.

Run	Factor			Total Phenolics, Uncorrected		
	X_1_	X_2_	X_3_	mg GAE g^−1^ DS ± SD (*n* = 2)		
1	−	−	−	6.54	±	0.72
2	+	−	−	5.76	±	0.07
3	−	+	−	9.04	±	0.52
4	+	+	−	5.26	±	0.59
5	−	−	+	7.82	±	0.38
6	+	−	+	4.84	±	0.16
7	−	+	+	7.64	±	0.61
8	+	+	+	6.27	±	0.03
9	−	0	0	7.66	±	0.16
10	+	0	0	6.30	±	0.25
11	0	−	0	5.12	±	0.62
12	0	+	0	7.68	±	0.98
13	0	0	−	7.63	±	0.03
14	0	0	+	6.39	±	1,10
15	0	0	0	7.03	±	0.72
16	0	0	0	5.93	±	0.38
17	0	0	0	7.82	±	0.17

## References

[1] Tchoné M., Bärwald G., Annemüller G., Fleischer L.-G. (2006). Separation and identification of phenolic compounds in Jerusalem artichoke (*Helianthus tuberosus* L.). Sci. Aliment..

[2] Jaiswal R., Deshpande S., Kuhnert N. (2011). Profiling the chlorogenic acids of *Rudbeckia hirta*, *Helianthus tuberosus*, *Carlina acaulis* and *Symphyotrichum novae-angliae* leaves by LC-MSn. Phytochem. Anal..

[3] Chen F., Long X., Liu Z., Shao H., Liu L. (2014). Analysis of phenolic acids of Jerusalem artichoke (*Helianthus tuberosus* L.) responding to salt-stress by liquid chromatography/tandem mass spectrometry. Sci. World J..

[4] Kapusta I., Krok E., Jamro D., Cebulak T., Kaszuba J., Salach R. (2013). Identification and quantification of phenolic compounds from Jerusalem artichoke (*Helianthus tuberosus* L.) Tubers. J. Food Agric. Environ..

[5] Yuan X., Gao M., Xiao H., Tan C., Du Y. (2012). Free radical scavenging activities and bioactive substances of Jerusalem artichoke (*Helianthus tuberosus* L.) leaves. Food Chem..

[6] Chen F., Long X., Yu M., Liu Z., Liu L., Shao H. (2013). Phenolics and antifungal activities analysis in industrial crop Jerusalem artichoke (*Helianthus tuberosus* L.) leaves. Ind. Crop. Prod..

[7] Petrova I., Petkova N., Ivanov I. (2016). Five edible flowers—Valuable source of antioxidants in human nutrition. Int. J. Pharmacogn. Phytochem. Res..

[8] Petkova N., Ivanov I., Denev P., Pavlov A. (2014). Bioactive substance and free radical scavenging activities of flour from Jerusalem artichoke (*Helianthus tuberosus* L.) Tubers—A comparative study. Türk Tarım Doğa Bilimleri.

[9] Sun P.-C., Liu Y., Yi Y.-T., Li H.-J., Fan P., Xia C.-H. (2015). Preliminary enrichment and separation of chlorogenic acid from *Helianthus tuberosus* L. leaves extract by macroporous resins. Food Chem..

[10] Pan L., Sinden M.R., Kennedy A.H., Chai H., Watson L.E., Graham T.L., Kinghorn A.D. (2009). Bioactive constituents of *Helianthus tuberosus* (Jerusalem artichoke). Phytochem. Lett..

[11] Lim T. (2015). *Helianthus* *tuberosus*. Edible Medicinal and Non Medicinal Plants.

[12] Yang L., He Q.S., Corscadden K., Udenigwe C.C. (2015). The prospects of Jerusalem artichoke in functional food ingredients and bioenergy production. Biotechnol. Rep..

[13] Zhang Q., Kim H.-Y. (2015). Antioxidant, anti-inflammatory and cytotoxicityon human lung epithelial A549 cells of Jerusalem artichoke (*Helianthus tuberosus* L.) Tuber. Korean J. Plant Res.

[14] Johansson E., Prade T., Angelidaki I., Svensson S.-E., Newson W.R., Gunnarsson I.B., Hovmalm H.P. (2015). Economically viable components from Jerusalem artichoke (*Helianthus tuberosus* L.) in a biorefinery concept. Int. J. Mol. Sci..

[15] Slimestad R., Seljaasen R., Meijer K., Skar S.L. (2010). Norwegian-grown Jerusalem artichoke (*Helianthus tuberosus* L.): Morphology and content of sugars and fructo-oligosaccharides in stems and tubers. J. Sci. Food Agric..

[16] Bach V., Clausen M.R., Edelenbos M. (2015). Production of Jerusalem artichoke (*Helianthus tuberosus* L.) and impact on inulin and phenolic compounds. Processing and Impact on Active Components in Food.

[17] Isabelle M., Lee B.L., Lim M.T., Koh W.-P., Huang D., Ong C.N. (2010). Antioxidant activity and profiles of common vegetables in Singapore. Food Chem..

[18] Sánchez-Rangel J.C., Benavides J., Heredia J.B., Cisneros-Zevallos L., Jacobo-Velázquez D.A. (2013). The Folin–Ciocalteu assay revisited: Improvement of its specificity for total phenolic content determination. Anal. Methods.

[19] Gunnarsson I.B., Svensson S.E., Johansson E., Karakashev D., Angelidaki I. (2014). Potential of Jerusalem artichoke (*Helianthus tuberosus* L.) as a biorefinery crop. Ind. Crops Prod..

[20] Long X.-H., Shao H.-B., Liu L., Liu L.-P., Liu Z.-P. (2016). Jerusalem artichoke: A sustainable biomass feedstock for biorefinery. Renew. Sustain. Energy Rev..

[21] Siatka T., Kašparová M. (2010). Seasonal variation in total phenolic and flavonoid contents and DPPH scavenging activity of *Bellis perennis* L. flowers. Molecules.

[22] Prat D., Wells A., Hayler J., Sneddon H., McElroy C.R., Abou-Shehada S., Dunn P.J. (2015). CHEM21 selection guide of classical-and less classical-solvents. Green Chem..

[23] Sepahpour S., Selamat J., Abdul Manap M.Y., Khatib A., Abdull Razis A.F. (2018). Comparative analysis of chemical composition, antioxidant activity and quantitative characterization of some phenolic compounds in selected herbs and spices in different solvent extraction systems. Molecules.

[24] Kim J.-W., Kim J.-K., Song I.-S., Kwon E.-S., Youn K.-S. (2013). Comparison of antioxidant and physiological properties of Jerusalem artichoke leaves with different extraction processes. J. Korean Soc. Food Sci. Nutr..

[25] Yuan X., Cheng M., Gao M., Zhuo R., Zhang L., Xiao H. (2013). Cytotoxic constituents from the leaves of Jerusalem artichoke (*Helianthus tuberosus* L.) and their structure–activity relationships. Phytochem. Lett..

[26] Sharma O.P., Bhat T.K. (2009). DPPH antioxidant assay revisited. Food Chem..

